# Adapting Evidence‐Based Practice Guidelines for Sedation, Analgesia, Withdrawal, and Delirium Assessment and Management in Critically Ill Children

**DOI:** 10.1155/ccrp/7830579

**Published:** 2026-06-12

**Authors:** Muneera Al-Jelaify, Gamal Hasan, Mohamad-Hani Temsah, Mohammed Almazyad, Ali Alhaboob, Majed Alabdulhafid, Fahad Alsohime, Abdullah Alturki, Waleed Albuali, Alanoud Almuhareb, Abdulaziz Alsoqati, Ivan D. Florez, Yasser S. Amer, Ayman Al-Eyadhy

**Affiliations:** ^1^ Corporate Department of Pharmacy Services, King Saud University Medical City, King Saud University, Riyadh, Saudi Arabia, ksu.edu.sa; ^2^ Faculty of Medicine, Assiut University, Assiut, Egypt, aun.edu.eg; ^3^ Sheikh Shakhbout Medical City, Abu Dhabi, UAE, ssmcabudhabi.ae; ^4^ Department of Pediatrics, College of Medicine, King Saud University, Riyadh, Saudi Arabia, ksu.edu.sa; ^5^ Pediatric Intensive Care Unit, King Saud University Medical City, King Saud University, Riyadh, Saudi Arabia, ksu.edu.sa; ^6^ Pediatric Critical Care, King Faisal Specialist Hospital & Research Center, Riyadh, Saudi Arabia, kfshrc.edu.sa; ^7^ Department of Pediatrics, College of Medicine, Imam Abdulrahman Bin Faisal University, Dammam, Saudi Arabia, iau.edu.sa; ^8^ King Fahd Hospital of the University, Al-Khobar, Saudi Arabia, iau.edu.sa; ^9^ Pharmaceutical Care Department, King Faisal Specialist Hospital & Research Center, Riyadh, Saudi Arabia, kfshrc.edu.sa; ^10^ Pediatric Intensive Care Unit, King Fahad Medical City, Riyadh, Saudi Arabia, kfmc.med.sa; ^11^ Department of Pediatrics, University of Antioquia, Medellin, Colombia, udea.edu.co; ^12^ School of Rehabilitation Science, McMaster University, Hamilton, Canada, mcmaster.ca; ^13^ Pediatric Intensive Care Unit, Clinica Las Americas, Medellin, Colombia; ^14^ Pediatrics Department, King Khalid University Hospital, King Saud University Medical City, King Saud University, Riyadh, Saudi Arabia, ksu.edu.sa; ^15^ Clinical Practice Guidelines and Quality Research Unit, Quality Management Department, King Saud University Medical City, King Saud University, Riyadh, Saudi Arabia, ksu.edu.sa; ^16^ Department of Internal Medicine, Ribeirão Preto Medical School, University of São Paulo (FMRP-USP), Ribeirão Preto, São Paulo, Brazil

**Keywords:** AGREE II, analgesia, children, critical care, evidence-based, guideline adaptation, healthcare quality, implementation, KSU-modified ADAPTE, patient safety

## Abstract

**Objectives:**

To adapt evidence‐based clinical practice guidelines (CPGs) for sedation, analgesia, withdrawal, and delirium assessment and management with a focus on early mobility in critically ill children admitted to a pediatric intensive care unit (PICU).

**Design:**

The panel included two groups: the guideline adaptation group (GAG), which consisted of two consultant pediatric intensivists, a clinical pharmacist, a senior nurse specialist, and a general pediatrician and a CPG methodologist, and an external review and consultation group, which included five consultant pediatric intensivists, a clinical pharmacist, and two CPG expert physicians. A formal methodology for CPG adaptation was followed and included three phases: set‐up, adaptation, and finalization.

**Results:**

The provided assessment tools and management algorithms focused on adequacy of sedation and analgesia, medication selection, dosing, initiation, and adjustment for both noninvasive and invasive mechanical ventilation; withdrawal assessment; weaning of sedation and analgesia; withdrawal management; delirium screening; delirium management; as well as early mobility facilitation. Implementation tools included behavioral scoring tool for sedation and pain, Withdrawal Assessment Tool with a management guide, screening score for delirium with a management guide, 3 clinical management algorithms for sedation management, analgesia management, and weaning process.

**Conclusions:**

Based on a systematic adaptation framework and the interdisciplinary and multidisciplinary approach, the present study formulated an adapted CPG with practically applicable implementation tools and management algorithms that might help standardize sedation, analgesia, and withdrawal assessment and management in critically ill children at the PICU. We recommend further studies to clarify the generalizability, impact of its implementation, and practices to overcome potential barriers.

## 1. Introduction

Sedation and analgesia are crucial elements in the management of critically ill patients, especially in those requiring invasive ventilation [1–3]. The main indications for the use of sedatives and analgesics are control of pain, anxiety and agitation, induction of amnesia, facilitation of invasive and noninvasive mechanical ventilation, prevention of endotracheal tube displacement and self‐harm, and reduction of metabolic demands [1, 4, 5].

Both under‐sedation and over‐sedation have a negative impact on patients’ therapeutic outcomes. Under‐sedation, for instance, can increase the risk of adverse events, such as displacement of catheters and self‐extubation. Over‐sedation, on the other hand, can lead to extubation failure and prolonged mechanical ventilation and length of stays [6–10]. Moreover, the prolonged use of sedatives and analgesics increases the risk of developing tolerance and withdrawal phenomena, which can significantly impact the overall healthcare costs [7, 11–14]. Therefore, reaching a balance while navigating through the daily management goals of critically ill children is essential [15].

These sedation challenges need effective monitoring and management of sedation and analgesia. In a survey of sedation and neuromuscular blocker practices, Playfor et al. found that written clinical guidelines for the sedation of critically ill children were only available in 45% of pediatric intensive care units (PICUs), while sedation is formally assessed in 40% of units among the 18 PICUs that were included in the study in the United Kingdom [16]. Local and international surveys have found that only 42%–55% of those who have sedation scoring systems use them on a daily basis [17, 18]. Moreover, there is heterogeneity among regions regarding the scoring system used. In North America, for instance, it has been reported that the State Behavioral Scale (SBS) was the most often used (22%), whereas in other countries, it is the COMFORT score (39%). Furthermore, significant variation in the practice of pharmacologic sedation was reported, and a common theme was frustration over inconsistent sedation goals [19–21].

On the other hand, delirium is another problem in critically ill children that is going underdiagnosed [22]. This is because signs and symptoms of delirium are overlooked by healthcare professionals and the difficulty in observing it in nonverbalizing patients [23–26]. Symptoms of delirium can be misidentified as pain, distress, or withdrawal syndrome. Screening for delirium is not widely implemented in PICUs despite the availability of assessment tools.

In addition to systematic screening, nonpharmacologic interventions are increasingly recognized as essential components of delirium prevention and management. These include environmental optimization, sleep promotion, family engagement, and structured delirium prevention measures (see Supporting Table S9: Delirium prevention measures), such as maintaining day–night orientation, minimizing noise and light during rest periods, clustering care activities, promoting a familiar environment, and reducing unnecessary restraints and invasive devices. Early mobility has emerged as an important supportive strategy in pediatric critical care, with evidence suggesting that structured mobilization may reduce delirium risk, shorten mechanical ventilation duration, and improve functional outcomes [27]. Although pediatric data remain limited compared to adults, available studies and expert consensus support the feasibility and safety of early mobility when integrated into multidisciplinary care bundles [26]. Accordingly, these nonpharmacologic strategies, including early mobility, were incorporated into the scope of the adapted guideline as part of a holistic approach to sedation, analgesia, withdrawal, and delirium care. Management protocols are potential tools to standardize clinical practices of sedation management. However, Curley et al. in a randomized clinical trial of protocoled sedation versus usual care found that the use of a nurse‐implemented, goal‐directed sedation protocol compared with usual care did not reduce the duration of mechanical ventilation [28]. Alternatively, Yang et al. reported a positive impact of sedation and analgesia protocol on the reduction of midazolam use but no other evident impact on other clinical outcomes such as length of stay or mechanical ventilation duration [29]. Hence, sedation, analgesia, withdrawal, and delirium assessment and management and early mobility facilitation among critically ill children have been identified as areas for improvement that can impact the patients ‘outcome and the overall quality of care in hospital settings.

Clinical practice guidelines (CPGs) are defined as statements that include recommendations intended to optimize patient care, which are informed by a systematic review of evidence and an assessment of the benefits and harms of alternative care options [30]. CPGs have been identified repeatedly as major tools for improving healthcare quality and safety [30–32]. Adaptation of CPGs is an efficient alternative to the development of CPGs “from the scratch,” i.e., CPG de novo development. Adaptation aims to avoid duplication of efforts to use the available resources in a cost‐effective manner and to encourage transcontextual customization of the CPG prepared for a different healthcare setting to reflect the local context and system [30–33].

To date, the published CPGs for sedation and analgesia in children admitted to PICUs are few. Hence, this paper presents an updated evidence‐based adapted CPG for sedation, analgesia, withdrawal, and delirium assessment and management and early mobility in critically ill children at the PICUs.

## 2. Methodology

An evidence‐based quality improvement project was initiated, aiming to adapt an evidence‐based CPG for the targeted scope in the Kingdom of Saudi Arabia. Interdisciplinary and structured multidisciplinary discussion meetings were held to identify the CPG adaptation process, its main sources, and the review process. Also, the study team identified the required training, education, dissemination, and pilot implementation plan. The King Saud University (KSU)–modified ADAPTE approach/methodology has been followed throughout the adaptation process, which consists of three phases and 24 steps [30, 34–36]. Figure S1 illustrates a summary of the KSU‐modified ADAPTE process for CPG adaptation.

### 2.1. Phase 1 (Set‐Up)

The topic of sedation and analgesia in the pediatric critical care setting was selected as a high‐priority health problem with considerable practice variation and a lack of national evidence–based CPGs.

An initial exploratory search for the relevant published CPGs was conducted. The guidelines adaptation group (GAG) was formulated at the outset, including the PICU clinical pharmacist, two consultant pediatric intensivists, and the PICU head nurse, in addition to the CPG expert methodologist with a background in general pediatrics. The preliminary search for published literature in this area revealed a scarcity of evidence and a very limited number of evidence‐based CPGs addressing this subject. The GAG has checked and confirmed the feasibility of CPG adaptation by retrieving published CPGs, identifying the required resources and skills, writing up the adaptation working plan, and declaring the conflicts of interest of all the members.

The adapted guideline is intended for use by PICU physicians, nurses, clinical pharmacists, and respiratory therapists, reflecting their shared roles in sedation, analgesia, withdrawal, and delirium assessment and management. Nursing representation within the GAG was provided by a senior PICU nurse specialist, with additional nursing input incorporated during external review and implementation planning.

### 2.2. Phase 2 (Adaptation)

In phase two, the GAG identified specific clinical questions using the PIPOH model (Table S1), relevant eligibility criteria, and a search strategy including a list of keywords. We searched eight bibliographic and CPG databases in addition to online libraries of relevant professional societies and conducted a systematic review following the Preferred Reporting Items for Systematic Reviews and Meta‐Analyses (PRISMA) [37].

### 2.3. Eligibility Criteria

Our eligibility criteria were as follows: (1) evidence‐based CPGs (i.e., any CPG with a clear and detailed documentation of the CPG development methodology in the Methods section); (2) English language; (3) original Source CPGs (de novo developed); (4) categorized as local, national, or international CPGs in the full‐text of the CPG; (5) published (between 1/1/2013 and 31/12/2018) 5 years before the project launching with the aim of identifying guidelines that had the most relevant and current evidence, and the search was repeated before the final paper submission to identify any new relevant CPG; and (6) published by an organization or group authorship in a CPG database, guideline developer website, or peer‐reviewed journal. The adaptation process used only the most updated version of each utilized Source CPG whenever available.

### 2.4. Search, Screen, and Selection of Sedation and Analgesia CPGs

We used literature searches of bibliographic databases (MEDLINE/PubMed and Google Scholar), EBSCO DynaMed Plus (USA), and CPG databases: the ECRI Institute Guidelines Trust, Guidelines International Network (GIN) International guideline library, Scottish Intercollegiate Guidelines Network (SIGN) (UK), National Institute of Health and Care Excellence (NICE) (UK), and the Australian National Health and Medical Research Council (NHMRC) (Australia). Keywords identified included the following: practice guidelines, guidelines, protocols, pathways, recommendations, pediatrics, children, critical care, PICU, sedation, analgesia, withdrawal, delirium, and early mobility.

The retrieved CPGs’ methodological quality was evaluated by four independent appraisers using the second version of the Appraisal of Guidelines for Research and Evaluation (AGREE II) Instrument. The clinician members of the GAG received training and education on CPG standards and appraisal tools (including the AGREE II) and were members of the CPG committee within the pediatrics department as part of the CPG adaptation program at KSU (25). AGREE II is a valid and reliable instrument with 23 items structured into six standardized domains and is considered the gold standard for quality assessment of CPGs [38, 39]. The GAG selected the high‐quality CPGs based on the superior ratings of the AGREE II domains (with a focus on the first overall assessment [or OA1]) if all elements of the sedation management are covered. The GAG decided to classify the appraised CPGs into low‐quality CPGs (OA1: less than 40%), moderate‐quality CPGs (OA1: 40%–59%), and high‐quality CPGs (OA1: more than 60%). Disagreements regarding eligibility decisions or AGREE II domain scores were resolved through structured consensus discussions and an informal voting process within the GAG, facilitated by the CPG methodologist.

Afterward, the GAG identified the relevant recommendations and discussed and revised them through successive structured multidisciplinary discussion meetings. We mapped all the recommendations from the source guidelines and checked how these recommendations overlap and the differences among them. Considering the AGREE II results, we brought the recommendations to a panel discussion to evaluate their differences and the quality of the evidence and the Source CPG. We discussed the feasibility and affordability of some of these recommendations in the context of the PICU and decided by consensus what would be the best recommendations for our context. Afterward, we drafted the final recommendations. Additional algorithms were modified or developed and added as implementation tools. Drafting the first version of the adapted CPG was the last step of this phase. Withdrawal and delirium were included within the search scope; however, stand‐alone pediatric CPGs addressing these domains were limited, and relevant recommendations were primarily identified within comprehensive sedation or critical care guidelines and position statements.

The PRISMA 2020 flow diagram was revised to ensure numerical consistency across all stages, with explicit differentiation between guidelines appraised using AGREE II and additional documents used solely for implementation support.

### 2.5. Phase 3 (Finalization)

In phase three, a full document of the first draft of the adapted CPG was finalized, including assessment of the key recommendations for acceptability and applicability in our setting, through a structured multidisciplinary discussion meeting by the multidisciplinary clinical members of the GAG. The adapted CPG draft was sent to a selected panel of external reviewers, including stakeholders and healthcare providers from relevant specialties who accepted the invitation to contribute. The panel consisted of pediatric intensivists, nurses, a clinical pharmacist, and a guideline methodologist involved in the assessment and management of sedation, analgesia, withdrawal, and delirium in the PICU. The feedback and suggestions of these reviewers were revised and discussed within the GAG and were reflected in a second, and then a final version of the adapted CPG with a set of CPG implementation (CPGI) tools was included in the final CPG full document, and several implementation strategies were identified and developed based on the key recommendations of the adapted CPG.

## 3. Results

### 3.1. Adaptation of the CPG

This work marks the 8^th^ CPG adaptation project at our PICU and pediatrics department as a part of our CPG adaptation program, as we had previous experience with the adaptation of several PICU CPGs [30, 40].

### 3.2. Adaptation Process

#### 3.2.1. Phase 1 (Set‐Up)

The multidisciplinary GAG with expertise in pediatric critical care, pharmacy, and/or CPGs was assembled, and the health topic was selected.

#### 3.2.2. Phase 2 (Adaptation)

We retrieved 1625 articles from databases and manual searches, which were further filtered based on the PIPOH elements and the eligibility criteria, leaving only four eligible records (Figure S2). Four Source CPGs for PICU sedation and analgesia were retrieved and critically appraised using the AGREE II Instrument following its standard domain scores using the calculation equation provided by the AGREE II user manual, and any disagreements were resolved by discussion [2, 28, 41, 42].

The results of the AGREE II assessment of the four Source CPGs are presented in Table S2. Two Source CPGs revealed superior ratings in OA1: European Society of Pediatric and Neonatal Intensive Care (ESPNIC) (position statement) 2016 and Nottingham University Hospitals NHS Trust PICU guideline (NUH‐NHS CPG) 2018. After revision and discussion of the recommendations of these four CPGs, the GAG decided to adapt the final CPG core recommendations from the ESPNIC position statement (which covered assessment only) and NUH‐NHS (which covered management of withdrawal only) CPGs. Moreover, an additional source, a care pathway that addressed the sedation and analgesia management among mechanically ventilated children, was utilized for adapting the implementation tools of our adapted CPG; however, this care pathway was not eligible for the AGREE II appraisal as it is not a formal CPG [3].

**FIGURE 1:**
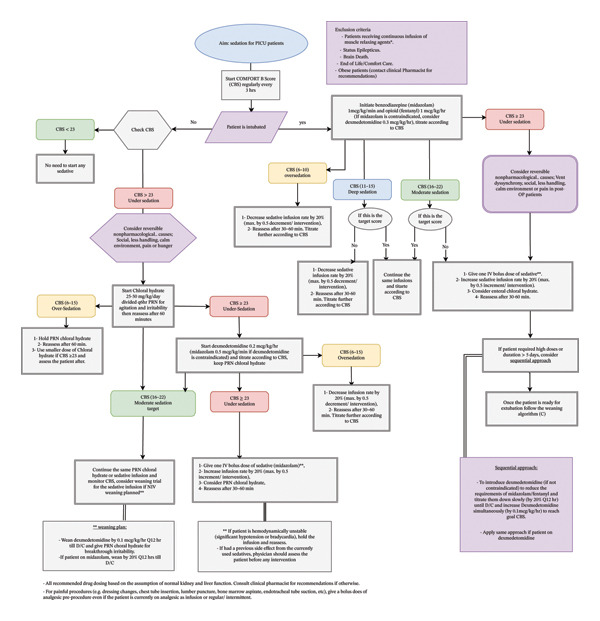
Algorithm A: Sedation management for PICU patients.

**FIGURE 2:**
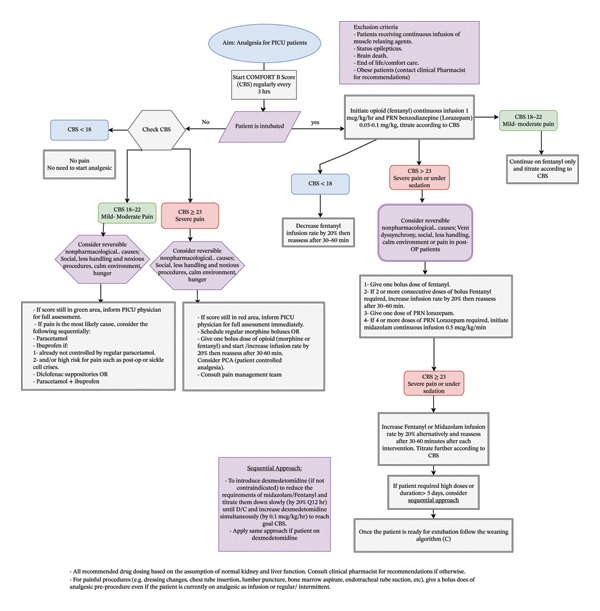
Algorithm B: Analgesia management for PICU patients.

**FIGURE 3:**
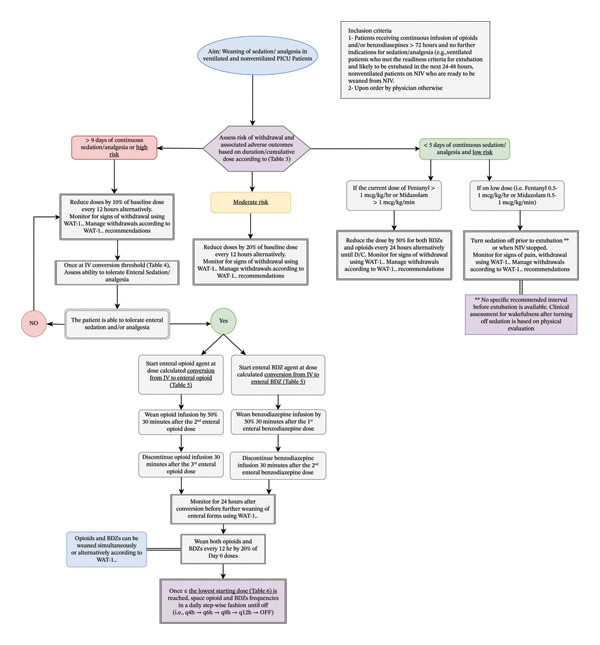
Algorithm C: Weaning of sedation/analgesia in PICU patients.

An initial draft was prepared with a set of CPGI tools including Modified–COMFORT Behavioral Score (CBS) for pain and sedation assessment (Supporting Table S3; Supporting Digital Content 1); three management algorithms: sedation management for children admitted to PICU, analgesia management for children admitted to PICU, and weaning of sedation/analgesia for children admitted to PICU (Figures 1, 2, and 3); Withdrawal Assessment Tool Version 1 (WAT‐1) with management guide (Supporting Table S4; Supporting Digital Content 1); risk categories for withdrawal and withdrawal‐associated adverse outcomes (Supporting Table S5; Supporting Digital Content 1); weaning intravenous (IV) sedation/analgesia to conversion thresholds (Supporting Table S6; Supporting Digital Content 1); conversion of opioids and benzodiazepines from IV infusion to enteral (Supporting Table S6; Supporting Digital Content 1); the lowest starting doses for per‐oral agents after which frequency can be weaned (Supporting Table S8; Supporting Digital Content 1); and delirium assessment and management using Cornell Assessment of Pediatric Delirium (CAPD) Score (Supporting Table S9; Supporting Digital Content 1). The CPGI tools included both formats; the electronic (computerized provider order) entry built in within our hospital electronic medical records system, in addition to a traditional paper format.

Moreover, a hospital‐wide policy and procedure that clarifies pediatric pain assessment, its assessment tools, management interventions, the process of sedatives and analgesics prescription, dispensing, administration, monitoring, and weaning within the PICU has been developed and approved through the pediatrics departmental quality team in collaboration with the hospital quality management department.

#### 3.2.3. Phase 3 (Finalization)

Following the external review step, the final CPG full document was formulated where a set of CPGI strategies was identified including the following: (i) leadership engagement and commitment, (ii) local clinical and quality champions, (iii) training and education, (iv) dissemination of the printed and electronic formats of the CPG and its implementation tools, (v) audit and feedback plan, and (vi) networking with relevant existing projects. The final version of the CPGI tools was included as well [30].

Table 1 summarizes the comparison between our adapted CPG and the included CPGs for sedation and analgesia in children in relation to the scope, used tools, assessment scores, strengths, weaknesses, study design, and used medications.

**TABLE 1:** Comparison between our adapted CPG and the included clinical practice guidelines for sedation and analgesia in children.

Parameter	KSUMC adapted CPG	Lamoureux et al. 2015 [23]	Fervers et al. [35]	Amer et al. [34]	Sedation and analgesia withdrawal clinical guidelines [2]	CHOP clinical pathways [3] revised 2020
*Scope*						
Target population	Children aged 1 month to 14 years who required noninvasive ventilation, invasive mechanical ventilation, postoperative analgesia, or procedural sedation, and they were started on intermittent or continuous infusions of sedatives and/or analgesics	Patients from 2 weeks to 17 years of age receiving invasive mechanical ventilation for acute airways and/or parenchymal lung disease	Pediatric or neonatal critical care nonverbal inpatients, with the age limits set from birth to 18 years	Mechanically ventilated PICU patients aged from 0 to 18 years receiving sedation for more than 24	Children and young people cared for at Nottingham Children Hospital	PICU mechanically ventilated patient
Goal	Assessment and management of sedation, pain, and withdrawal syndrome	Assessment and management of sedation and withdrawal syndrome	Assessment of pain, sedation, IWS, and delirium	Assessment and management of sedation and withdrawal syndrome	Assessment and management of withdrawal syndrome	Assessment and management of sedation, pain, and withdrawal syndrome

*Tools*						
	Table S2: CBS for pain and sedation assessment Figure S1: Clinical algorithm for sedation management for children admitted in PICU Figure S2: Clinical algorithm for analgesia management for children admitted in PICU Figure S3: Clinical algorithm for weaning of sedation/analgesia for children admitted in PICU. Table S3: WAT‐1 Table S4: Risk categories for withdrawal and withdrawal‐associated adverse outcomes Table S5: Weaning IV sedation/analgesia to conversion thresholds Table S6: Conversion of opioids and benzodiazepines form intravenous infusion to enteral Table S7: Lowest starting doses for PO agents after which frequency can be weaned. Table S8: Delirium assessment and management using CAPD score	Supplement 2: RESTORE nurse‐implemented goal‐directed comfort algorithm for assessment and management of sedation and withdrawal syndrome	Table 1: Definitions of pain, distress, withdrawal syndrome and delirium Table 2: Panel of behavioral instruments specific to pediatric critical care Table 3: Pain: summary of recommended assessment tools for neonates and critically ill children Table 4: Sedation: summary of recommended assessment tools for critically ill children Table 5: IWS and delirium: summary of recommended assessment tools for critically ill children	Figure 1: Nurse‐driven sedation protocol	Sedation weaning flow chart. Sedation and analgesia withdrawal plan and scoring chart	Pathways: sedation and analgesia for patient with an expected length of intubation < 48 hours ‐ Sedation and analgesia for patients < 50 kg with an expected length of intubation ≥ 48 h ‐ Sedation and analgesia for patients ≥ 50 kg with an expected length of intubation ≥ 48 h ‐ PICU sedation/analgesia weaning

*Assessment scores*						
Pain	CBS	FLACC (nonverbal, 0–6 years), INRS (nonverbal cognitively impaired, ≥ 6 years), and WBFPS (verbal ≥ 3 years)	Neonates: PIPP, PIPP‐ revised, COMFORT new Infants and children: COMFORT‐B, FLACC, MAPS	Not included	Not included	r‐FLACC (term NB – 7 years), FACES (≥ 3 years, able to self‐report), numeric rating scale (> 5 years)
Sedation	CBS	SBS	COMFORT scale, COMFORT‐B, SBS.	COMFORT‐B	Not included	SBS
Withdrawal	WAT‐1	WAT‐1	WAT‐1, SOS, p CAM‐ICU, CAPD, SOS‐PD	WAT‐1	WAT‐1	WAT‐1
Delirium	CAPD	Not included		Not included	Not included	CAPD

*Strengths*						
	(1) Included assessment and management for sedation, pain, delirium, and withdrawal symptoms (2) Included invasively and noninvasively ventilated patients, postoperative and other categories who need analgesia (3) Nurse‐driven. (4) Included ERT (5) MDR on a daily basis to discuss: patient’s course of illness (acute, titration, or weaning phase); CBS target score, ERT, weaning plan when ready and a written sedation and/or analgesia weaning plan when transferred out of the PICU (6) It is a comprehensive CPG (7) Patients receiving neuromuscular blockade are excluded, since assessment scores will not be reliable (8) Considered the nonpharmacological options for sedation and pain management	(1) Included assessment and management of sedation (2) Included assessment and management of withdrawal symptoms (3) Pre/postprotocol data. (4) Nurse‐driven. (5) Included ERT. (6) MDR on a daily basis to discuss: patient’s course of illness (acute, titration, or weaning phase); SBS target score, ERT, weaning plan when ready and a written sedation weaning plan when transferred out of the PICU	(1) For each score, they provided: age range, variables assessed, score range (cutoff point), reliability data, forms of validity established, clinical utility, and grade of evidence (2) Included delirium (3) Covered invasively and noninvasively ventilated patients	(1) Included assessment and management of sedation (2) Included assessment and management of withdrawal symptoms (3) Pre/postprotocol data (4) Nurse‐driven (5) ERT	(1) Included assessment and management of withdrawal symptoms	(1) Patients receiving neuromuscular blockade are excluded (2) Delirium is considered (3) Considered the nonpharmacological options for sedation and pain management (4) Detailed pathways

*Weakness*						
	None	(1) Did not include noninvasively ventilated patients (2) Did not include delirium (3) Patients receiving neuromuscular blockade are included; pain/agitation was judged to be present when a patient demonstrated a ≥ 20% increase in HR or BP when stimulated	Did not include management as it was only assessment.	(1) Did not include pain assessment and management (2) Did not include noninvasively ventilated patients (3) Did not include delirium (4) Patients under neuromuscular blockade were not excluded (5) Although it is a nurse‐ driven protocol, yet nurses’ actions are limited to the group of adequate sedation (scores between 11 and 17). In case of deep sedation (scores between 7 and 11), modifications had to be validated by the physician	(1) Included only assessment and management of withdrawal symptoms	1‐ It is a pathway based on single‐center experience

*Design*						
	CPG	Protocol	Position statement	Protocol	CPG	Clinical pathway

*Medications used*						
Sedatives	Midazolam, dexmedetomidine, chloral hydrate, lorazepam, and diazepam	Midazolam, dexmedetomidine, propofol, clonidine, pentobarbital, and ketamine	NA	Midazolam and ketamine	NA	Lorazepam, dexmedetomidine, midazolam, pentobarbital, and ketamine
Analgesics	Fentanyl, morphine, NSAIDs (ibuprofen, diclofenac), and paracetamol	Morphine and fentanyl	NA	Sufentanil	NA	Fentanyl and hydromorphone
Withdrawal treatment	Lorazepam, diazepam, morphine, methadone, and clonidine	Methadone and clonidine	NA	Methadone, hydroxyzine, clonidine, and long half‐life benzodiazepines or neuroleptics	Morphine, lorazepam, diazepam, buccal midazolam, and clonidine	Morphine, hydromorphone, methadone, and oxycodone

*Note:* COMFORT‐B: COMFORT behavior scale.

Abbreviations: BP = blood pressure; CAPD = Cornell Assessment of Pediatric Delirium; CBS = COMFORT Behavioral Score; CPG = clinical practice guideline; ERT = extubation readiness test; ESPNIC = European Society of Pediatric and Neonatal Intensive Care; FLACC = Face, Legs, Activity, Cry, Consolability; HR = heart rate; INRS = individualized numeric rating scale; IWS = iatrogenic withdrawal syndrome; MAPS = Multidimensional Assessment of Pain Scale; MDR = multidisciplinary round; NA = not applicable; NHS = United Kingdom National Health Service; pCAM‐ICU = pediatric confusion assessment method–intensive care unit; PICU = pediatric intensive care unit; PIPP = Premature Infant Pain Profile; r‐FLACC = Revised Face, Legs, Activity, Cry, Consolability; RESTORE = Randomized Evaluation of Sedation Titration for Respiratory Failure; SBS = State Behavioral Scale; SOS = Sophia Observation Withdrawal Symptoms Scale; SOS‐PD = Sophia Observation Withdrawal Symptoms Pediatric Delirium Scale; WAT‐1 = Withdrawal Assessment Tool Version 1; WBFPS = Wong–Baker Faces Pain Scale.

### 3.3. Key Recommendations

Table 2 highlights the key recommendations of the adapted CPG.

**TABLE 2:** Summary of key recommendations of the adapted CPG for sedation and analgesia management in critically ill infants and children.







## 4. Discussion

Both under‐sedation and over‐sedation are known to adversely affect clinical outcomes in critically ill children. Under‐sedation may increase the risk of adverse events such as accidental device removal and self‐extubation, whereas over‐sedation has been associated with delayed extubation, prolonged mechanical ventilation, and increased length of stay [6–8, 43]. These risks underscore the importance of standardized, objective, and balanced approaches to sedation and analgesia in the PICU.

The four CPGs identified, reviewed, and appraised during the adaptation process were predominantly consensus‐based and lacked a formal guideline development methodology, with the exception of the ESPNIC position statement, which included a comprehensive literature review. When assessed using the AGREE II instrument, only the ESPNIC position statement and NUH‐NHS CPG demonstrated acceptable quality in the rigor of development domain. To address these limitations, we employed the KSU‐modified ADAPTE methodology, which provides a structured and transparent framework for guideline adaptation, including systematic searching and selection of Source CPGs, formal quality appraisal using AGREE II, external review, and the development of practical implementation tools [30, 34–36].

The primary objective of the present work was to adapt existing CPGs to standardize the assessment and care of sedation, analgesia, withdrawal, and delirium in critically ill children using validated tools and structured management pathways. Objective assessment formed a central component of this approach. Accordingly, we selected the CBS to assess both sedation and pain, the WAT‐1 to evaluate opioid and benzodiazepine withdrawal, and the CAPD to screen for delirium. The use of the CBS for both pain and sedation assessment in our guideline was informed by its established validity for assessing distress, pain‐related behaviors, and sedation depth in critically ill children across a broad age range, as supported by prior literature.

In our setting, CBS underwent local pilot implementation to assess feasibility, interrater consistency, and clinical usability rather than formal psychometric validation. We did not perform a full validation study (e.g., criterion validity or sensitivity analyses). In contrast, other published guidelines and studies have employed multiple instruments, including the SBS, Multidisciplinary Assessment of Pain Scale (MAPS), and opioid benzodiazepine Withdrawal Assessment Scale (WAS), some of which lack robust validation across pediatric populations [44, 45].

Although Keogh et al.’s guideline was not included among the Source CPGs formally appraised using the AGREE II instrument and therefore did not directly inform the adaptation process, it is referenced here as a contextual comparator. Keogh et al. represent one of the most widely cited implementation‐focused pediatric PICU sedation guidelines and provide a useful perspective on feasibility and operationalization. Comparisons with Keogh et al. are therefore intended to contextualize implementation considerations rather than to indicate a foundational role in evidence synthesis or guideline development.

Keogh et al. reported reductions in sedative exposure following guideline implementation, although no significant impact on duration of mechanical ventilation or length of stay was observed, and overall feasibility was demonstrated [4]. In the present work, observational screening and early clinical use indicated that the adapted guideline was operationally feasible across PICU patients with diverse diagnoses and indications for sedation and analgesia, without formal evaluation of clinical outcomes.

Our PICU is a tertiary mixed medical–surgical unit (excluding cardiac surgery) with a broad admission profile. Unlike some prior implementation studies that focused primarily on mechanically ventilated children, our adapted guideline targets a wider population, including infants and children receiving invasive or noninvasive ventilation, procedural sedation, or intermittent analgesia. This broader scope enhances the generalizability of the adapted CPG across varied PICU settings and clinical scenarios. To further enhance applicability across diverse healthcare systems, the adapted guideline was intentionally designed to be flexible and modular. Implementation considerations for resource‐limited PICUs and settings with variable drug availability were explicitly addressed through prioritization of core assessment principles, use of commonly available medications, and algorithm‐based decision support that can be adapted when full implementation is not feasible. In keeping with established guideline adaptation methodologies, local multidisciplinary review and contextual tailoring are emphasized as essential steps prior to implementation. These considerations strengthen the generalizability and practical relevance of the guideline across a wide range of clinical environments.

A range of tools has been used internationally to assess pain, sedation, withdrawal, and delirium in children [46]. For pain assessment, commonly used instruments include FLACC, INRS, and r‐FLACC, while multiple sedation scales are also reported. The CBS has been validated for assessing sedation in both mechanically ventilated and spontaneously breathing critically ill children [46–52]. In the present guideline, CBS was adapted with predefined cutoff points to classify sedation depth and pain severity, allowing a single instrument to be used for both domains. During observational screening, CBS‐based assessments aligned with clinical impressions and other scales used in routine practice. The use of a single, validated tool for both pain and sedation assessment may simplify workflow and facilitate guideline implementation.

The WAT‐1 has demonstrated high validity, sensitivity, and specificity for detecting withdrawal in children [21], supporting its selection for withdrawal assessment in the adapted guideline. Delirium screening remains underutilized in PICUs despite the availability of validated instruments. Several tools have been validated across pediatric age groups, including PAED, pCAM‐ICU, and CAPD, with CAPD demonstrating favorable diagnostic performance across a wide age range [48–50]. Consistent with the ESPNIC position statement recommendations, CAPD was selected as the delirium screening instrument in the adapted guideline [42].

Management algorithms are integral to effective guideline implementation, translating recommendations into actionable bedside steps. In this work, three algorithms were developed to guide sedation management, analgesia management, and opioid/benzodiazepine weaning (Figures 1, 2, and 3), in addition to a structured table addressing delirium assessment and care (Supporting Digital Content Table S9). These tools were designed to support consistent clinical decision‐making across ventilated and nonventilated patients.

The adapted guideline emphasizes standardized pharmacologic strategies using commonly available agents, including opioids (fentanyl and morphine), benzodiazepines (midazolam and diazepam), and dexmedetomidine, while encouraging opioid‐sparing approaches such as acetaminophen and nonsteroidal anti‐inflammatory drugs for mild pain. Compared to previously published implementation‐focused guidelines, the adapted CPG provides clearer links between pain scores and pharmacologic choices. Additional considerations included recommendations for preprocedural analgesia using bolus dosing of existing infusions and structured approaches to transitioning from IV to enteral therapy during weaning.

For weaning of sedatives and analgesics, gradual dose reductions of 10%–20% per day were recommended, consistent with existing literature. Unlike some prior guidelines that rely primarily on clinical response or gastrointestinal function, the adapted CPG incorporates withdrawal risk stratification to guide the pace of weaning, aiming to minimize withdrawal symptoms while maintaining patient comfort.

The adapted CPG incorporates a phased dissemination strategy including staff education and orientation, consistent with established approaches to guideline uptake. However, formal evaluation of implementation outcomes and clinical impact was beyond the scope of the present manuscript and will be reported separately with dedicated analysis.

The adapted guideline presents stepwise key recommendations addressing sedation, analgesia, withdrawal, and delirium assessment and care in critically ill infants and children. Limitations of CBS are acknowledged, particularly in children with severe hypotonia, those receiving neuromuscular blockade, or in specific clinical contexts where supporting clinical judgment is required. Future directions align with Society of Critical Care Medicine recommendations, emphasizing ongoing monitoring with validated tools, protocolized care, and integration of nonpharmacologic interventions to optimize patient comfort and outcomes [53, 54]. Similar to Di Nardo et al., we had successful implementation of a PICU liberation bundle, prioritizing delirium screening and treatment, and sedation and early mobilization were feasible and safe even in a tertiary PICU that is the first to be reported in our region [55].

In conclusion, this work presents a systematically adapted, interdisciplinary CPG supported by practical implementation tools and management algorithms. The adapted guideline aims to address existing gaps in standardized assessment and care for sedation, analgesia, withdrawal, and delirium in critically ill children and may support national standardization efforts. Further evaluation of implementation fidelity and clinical impact is warranted in future studies.

### 4.1. Gaps in Knowledge and Research Opportunities

Multicenter studies utilizing the adapted guidelines are highly recommended to further clarify the safety and efficacy of sedation and analgesia agents for critically ill children in the PICU.

**Nomenclature**
ADAPTE: The original ADAPTE method refers to the guideline adaptation manual resource toolkit Version 2 (by the Adaptation Working Group, Guidelines International Network and the former ADAPTE collaboration)
AGREE II: Appraisal of Guidelines for Research and Evaluation (Second Version) Instrument
CPG: Clinical practice guideline
CBS: COMFORT Behavioral Score
GAG: Guideline (CPG) adaptation group
KSU‐modified ADAPTE: One of the formal adaptation methodologies for guidelines modified from the original ADAPTE (by the team at KSU or King Saud University)
MAPS: Multidisciplinary Assessment of Pain Scale
PICU: Pediatric intensive care unit
PRISMA: Preferred Reporting Items for Systematic Reviews and Meta‐Analyses
WAS: Withdrawal Assessment Scale
WAT‐1: Withdrawal Assessment Tool Version 1

## Author Contributions

Muneera Al Jelaify, Gamal Hasan, Yasser S. Amer, and Abdulaziz Alsoqati conceptualized and designed the study. Muneera Al Jelaify and Gamal Hasan contributed to the data collection, the systematic review of CPGs, and critical appraisal using the AGREE II. Muneera Al Jelaify, Gamal Hasan, and Yasser S. Amer have written the first draft of the manuscript. Abdulaziz Alsoqati supervised the study overall and reviewed the final version of this manuscript as a corresponding author.

## Funding

This study was supported by the Ongoing Research Funding Program (ORF‐2026‐1383), King Saud University, Riyadh, Saudi Arabia.

## Disclosure

All authors have made substantial contributions and gave final approval for the conception, drafting, and final version of this manuscript. All authors have critically reviewed the recommendations as content experts and approved the final version of the manuscript.

## Conflicts of Interest

The authors declare no conflicts of interest.

## Supporting Information

Additional supporting information can be found online in the Supporting Information section.

## Supporting information


**Supporting Information** The Supporting Information provides the assessment tools, scoring systems, and implementation aids used in the adapted CPG. Supporting Tables S1–S9 include the PIPOH model guiding question formulation; AGREE II domain scores for the source guidelines; the Modified‐CBS; WAT‐1; risk categorization tables, dosing, and conversion thresholds for sedation and analgesia weaning; and the CAPD delirium assessment and management guide. These supporting files are intended to support the implementation of the adapted guideline in clinical practice. Supporting Table S1; Supporting Digital Content 1: Health/Clinical Questions (PIPOH Model) outlining the clinical questions that guided the adaptation process. Supporting Table S2; Supporting Digital Content 1: AGREE II standardized domain scores for sedation and analgesia for critically ill children in PICU; AGREE II standardized domain scores for each Source CPG included in the appraisal. Supporting Table S3: Modified‐CBS for pain and sedation assessment; used for assessing pain and sedation in critically ill children. Supporting Table S4: WAT‐1; used for monitoring opioid and benzodiazepine withdrawal symptoms. Supporting Table S5: Risk categories for withdrawal, including definitions and associated adverse outcomes. Supporting Table S6: (Weaning IV sedation/analgesia to conversion thresholds): Criteria for transitioning from IV sedation/analgesia to conversion thresholds during the weaning process. Supporting Table S7: Conversion of opioids and benzodiazepines from IV infusion to enteral; used to guide switching opioids and benzodiazepines from IV infusion to enteral formulations. Supporting Table S8: Lowest starting doses for PO agents after which frequency can be weaned: recommended lowest starting doses for oral agents to support safe and structured dose weaning. Supporting Table S9: Delirium assessment and management using CAPD score. Figure S1. Summary of the KSU‐modified ADAPTE process for CPG adaptation. Figure S2: PRISMA 2020 flow diagram for new systematic reviews which included searches of databases and registers only.

## Data Availability

The data that support the findings of this study are available in the Supporting Material of this article.
