# Supplementary material for: Adapting Evidence‐Based Practice Guidelines for Sedation, Analgesia, Withdrawal, and Delirium Assessment and Management in Critically Ill Children
Source: Crit Care Res Pract. 2026 Jun 12;2026:7830579. doi: 10.1155/ccrp/7830579 (PMC13263535; doi:10.1155/ccrp/7830579)
Supplement: Supplementary file 1 — Supporting Information The Supporting Information provides the assessment tools, scoring systems, and implementation aids used in the adapted CPG. Supporting Tables S1–S9 include the PIPOH model guiding question formulation; AGREE II domain scores for the source guidelines; the Modified‐CBS; WAT‐1; risk categorization tables, dosing, and conversion thresholds for sedation and analgesia weaning; and the CAPD delirium assessment and management guide. These supporting files are intended to support the implementation of the adapted guideline in clinical practice. Supporting Table S1; Supporting Digital Content 1: Health/Clinical Questions (PIPOH Model) outlining the clinical questions that guided the adaptation process. Supporting Table S2; Supporting Digital Content 1: AGREE II standardized domain scores for sedation and analgesia for critically ill children in PICU; AGREE II standardized domain scores for each Source CPG included in the appraisal. Supporting Table S3: Modified‐CBS for pain and sedation assessment; used for assessing pain and sedation in critically ill children. Supporting Table S4: WAT‐1; used for monitoring opioid and benzodiazepine withdrawal symptoms. Supporting Table S5: Risk categories for withdrawal, including definitions and associated adverse outcomes. Supporting Table S6: (Weaning IV sedation/analgesia to conversion thresholds): Criteria for transitioning from IV sedation/analgesia to conversion thresholds during the weaning process. Supporting Table S7: Conversion of opioids and benzodiazepines from IV infusion to enteral; used to guide switching opioids and benzodiazepines from IV infusion to enteral formulations. Supporting Table S8: Lowest starting doses for PO agents after which frequency can be weaned: recommended lowest starting doses for oral agents to support safe and structured dose weaning. Supporting Table S9: Delirium assessment and management using CAPD score. Figure S1. Summary of the KSU‐modified ADAPTE process for CPG adaptat [file CCRP-2026-7830579-s001.zip › Figure S1 Main adaptation.docx]

**Figure S1. Summary of the KSU Modified ADAPTE process for CPG adaptation**

AGREE: Appraisal of Guidelines for Research and Evaluation; CPG: clinical practice guideline; CPGI: clinical practice guideline implementation; KSU: King Saud University; PIPOH: patient population, intervention, professionals, outcomes, healthcare settings.
