# Supplementary material for: Adapting Evidence‐Based Practice Guidelines for Sedation, Analgesia, Withdrawal, and Delirium Assessment and Management in Critically Ill Children
Source: Crit Care Res Pract. 2026 Jun 12;2026:7830579. doi: 10.1155/ccrp/7830579 (PMC13263535; doi:10.1155/ccrp/7830579)
Supplement: Supplementary file 1 — Supporting Information The Supporting Information provides the assessment tools, scoring systems, and implementation aids used in the adapted CPG. Supporting Tables S1–S9 include the PIPOH model guiding question formulation; AGREE II domain scores for the source guidelines; the Modified‐CBS; WAT‐1; risk categorization tables, dosing, and conversion thresholds for sedation and analgesia weaning; and the CAPD delirium assessment and management guide. These supporting files are intended to support the implementation of the adapted guideline in clinical practice. Supporting Table S1; Supporting Digital Content 1: Health/Clinical Questions (PIPOH Model) outlining the clinical questions that guided the adaptation process. Supporting Table S2; Supporting Digital Content 1: AGREE II standardized domain scores for sedation and analgesia for critically ill children in PICU; AGREE II standardized domain scores for each Source CPG included in the appraisal. Supporting Table S3: Modified‐CBS for pain and sedation assessment; used for assessing pain and sedation in critically ill children. Supporting Table S4: WAT‐1; used for monitoring opioid and benzodiazepine withdrawal symptoms. Supporting Table S5: Risk categories for withdrawal, including definitions and associated adverse outcomes. Supporting Table S6: (Weaning IV sedation/analgesia to conversion thresholds): Criteria for transitioning from IV sedation/analgesia to conversion thresholds during the weaning process. Supporting Table S7: Conversion of opioids and benzodiazepines from IV infusion to enteral; used to guide switching opioids and benzodiazepines from IV infusion to enteral formulations. Supporting Table S8: Lowest starting doses for PO agents after which frequency can be weaned: recommended lowest starting doses for oral agents to support safe and structured dose weaning. Supporting Table S9: Delirium assessment and management using CAPD score. Figure S1. Summary of the KSU‐modified ADAPTE process for CPG adaptat [file CCRP-2026-7830579-s001.zip › Figure S2 PRISMA_2020_flow_diagram (1).docx]

**Identification of studies via databases and registers**

Records removed *before screening*:

Duplicate records removed (n = 617)

Records marked as ineligible by automation tools (n = 0)

Records removed for other reasons (n = 0)

Records identified from*:

Databases (n = 1020)

Registers (n = 602)

**Identification**

Records screened

(n = 1005)

Records excluded**

(n = 995)

Reports sought for retrieval

(n = 0)

Reports not retrieved

(n = 995)

**Screening**

Reports excluded:

- Irrelevant studies (n = 411)

- Reviews (n = 210)

- Pediatrics, non-critically ill (n = 270)

- Sedation only (n = 106)

Reports assessed for eligibility

(n = 1005)

Studies included in review

(n = 4)

Reports of included studies

(n = 4)

**Included**

*Consider, if feasible to do so, reporting the number of records identified from each database or register searched (rather than the total number across all databases/registers).

**If automation tools were used, indicate how many records were excluded by a human and how many were excluded by automation tools.

*From:*  Page MJ, McKenzie JE, Bossuyt PM, Boutron I, Hoffmann TC, Mulrow CD, et al. The PRISMA 2020 statement: an updated guideline for reporting systematic reviews. BMJ 2021;372:n71. doi: 10.1136/bmj.n71

For more information, visit: <http://www.prisma-statement.org/>
