# Supplementary material for: Adapting Evidence‐Based Practice Guidelines for Sedation, Analgesia, Withdrawal, and Delirium Assessment and Management in Critically Ill Children
Source: Crit Care Res Pract. 2026 Jun 12;2026:7830579. doi: 10.1155/ccrp/7830579 (PMC13263535; doi:10.1155/ccrp/7830579)
Supplement: Supplementary file 1 — Supporting Information The Supporting Information provides the assessment tools, scoring systems, and implementation aids used in the adapted CPG. Supporting Tables S1–S9 include the PIPOH model guiding question formulation; AGREE II domain scores for the source guidelines; the Modified‐CBS; WAT‐1; risk categorization tables, dosing, and conversion thresholds for sedation and analgesia weaning; and the CAPD delirium assessment and management guide. These supporting files are intended to support the implementation of the adapted guideline in clinical practice. Supporting Table S1; Supporting Digital Content 1: Health/Clinical Questions (PIPOH Model) outlining the clinical questions that guided the adaptation process. Supporting Table S2; Supporting Digital Content 1: AGREE II standardized domain scores for sedation and analgesia for critically ill children in PICU; AGREE II standardized domain scores for each Source CPG included in the appraisal. Supporting Table S3: Modified‐CBS for pain and sedation assessment; used for assessing pain and sedation in critically ill children. Supporting Table S4: WAT‐1; used for monitoring opioid and benzodiazepine withdrawal symptoms. Supporting Table S5: Risk categories for withdrawal, including definitions and associated adverse outcomes. Supporting Table S6: (Weaning IV sedation/analgesia to conversion thresholds): Criteria for transitioning from IV sedation/analgesia to conversion thresholds during the weaning process. Supporting Table S7: Conversion of opioids and benzodiazepines from IV infusion to enteral; used to guide switching opioids and benzodiazepines from IV infusion to enteral formulations. Supporting Table S8: Lowest starting doses for PO agents after which frequency can be weaned: recommended lowest starting doses for oral agents to support safe and structured dose weaning. Supporting Table S9: Delirium assessment and management using CAPD score. Figure S1. Summary of the KSU‐modified ADAPTE process for CPG adaptat [file CCRP-2026-7830579-s001.zip › Table S2 AGREE II standardized domain scores for sedation and analgesia.docx]

**Table S2. AGREE II standardized domain scores for sedation and analgesia for critically ill children in PICU**

| **CPGs/ AGREE II Domains-standardized scores (%)** | **RESTORE 2015**^23^ | **ESPNIC** **2016**^35^ | **Dreyfus**  **2017**^34^ | **NHS**  **2018**^2^ |
| --- | --- | --- | --- | --- |
| **Domain 1. Scope and Purpose**  **Items 1-3:** Objectives; Health question(s); Population (patients, public, etc.). | 48% | 85% | 43% | 32% |
| **Domain 2. Stakeholder Involvement**  **Items 4-6:** Group Membership; Target population preferences and views; Target users. | 32% | 68% | 23% | 36% |
| **Domain 3. Rigour of development**  **Items 7-14:** Search methods; Evidence selection criteria; Strengths and limitations of the evidence; Formulation of recommendations; Consideration of benefits and harms; Link between recommendations and evidence; External review; Updating procedure. | 2% | 74% | 10% | 10% |
| **Domain 4. Clarity and presentation**  **Items 15-17:** Specific and unambiguous recommendations; Management options; Identifiable key recommendations | 62% | 100% | 54% | 56% |
| **Domain 5. Applicability**  **Items 18-21:** Facilitators and barriers to application; Implementation advice/ tools; Resource implications; Monitoring/ auditing criteria | 36% | 47% | 33% | 15% |
| **Domain 6. Editorial independence**  **Items 22, 23:** Funding body; Competing interests | 15% | 100% | 40% | 0% |
| **Overall Assessment 1** (Overall quality) | 23% | 79% | 23% | 42% |
| **Overall Assessment 2** (Recommend the CPG for use by the four appraisers) | Yes (n=0);  Yes, with modifications (n=2);  No (n=2). | Yes (n=1);  Yes, with modifications (n=3);  No (n=0). | Yes (n=0);  Yes, with modifications (n=0);  No (n=4). | Yes (n=0);  Yes, with modifications (n=3);  No (n=1). |
| This table uses the AGREE II Domain Score Color Coding proposed by Amer YS, Titi MA, Godah MW, et al.  Low Quality: RED <40%, Moderate Quality: YELLOW 40-59%, High Quality: GREEN ≥60%. | | | | |
