# Supplementary material for: Adapting Evidence‐Based Practice Guidelines for Sedation, Analgesia, Withdrawal, and Delirium Assessment and Management in Critically Ill Children
Source: Crit Care Res Pract. 2026 Jun 12;2026:7830579. doi: 10.1155/ccrp/7830579 (PMC13263535; doi:10.1155/ccrp/7830579)
Supplement: Supplementary file 1 — Supporting Information The Supporting Information provides the assessment tools, scoring systems, and implementation aids used in the adapted CPG. Supporting Tables S1–S9 include the PIPOH model guiding question formulation; AGREE II domain scores for the source guidelines; the Modified‐CBS; WAT‐1; risk categorization tables, dosing, and conversion thresholds for sedation and analgesia weaning; and the CAPD delirium assessment and management guide. These supporting files are intended to support the implementation of the adapted guideline in clinical practice. Supporting Table S1; Supporting Digital Content 1: Health/Clinical Questions (PIPOH Model) outlining the clinical questions that guided the adaptation process. Supporting Table S2; Supporting Digital Content 1: AGREE II standardized domain scores for sedation and analgesia for critically ill children in PICU; AGREE II standardized domain scores for each Source CPG included in the appraisal. Supporting Table S3: Modified‐CBS for pain and sedation assessment; used for assessing pain and sedation in critically ill children. Supporting Table S4: WAT‐1; used for monitoring opioid and benzodiazepine withdrawal symptoms. Supporting Table S5: Risk categories for withdrawal, including definitions and associated adverse outcomes. Supporting Table S6: (Weaning IV sedation/analgesia to conversion thresholds): Criteria for transitioning from IV sedation/analgesia to conversion thresholds during the weaning process. Supporting Table S7: Conversion of opioids and benzodiazepines from IV infusion to enteral; used to guide switching opioids and benzodiazepines from IV infusion to enteral formulations. Supporting Table S8: Lowest starting doses for PO agents after which frequency can be weaned: recommended lowest starting doses for oral agents to support safe and structured dose weaning. Supporting Table S9: Delirium assessment and management using CAPD score. Figure S1. Summary of the KSU‐modified ADAPTE process for CPG adaptat [file CCRP-2026-7830579-s001.zip › Table S3 CBS.docx]

**Table S3: Modified- COMFORT Behavioral Score (CBS)**

**For Pain & Sedation Assessment**

**Is the patient intubated: □ Yes □ No Date: ……………………………**

**Goal of Assessment: □ Sedation (Intubated) □ Sedation (non- invasively ventilated, NIV) □ Analgesia □ Routine screening of pain**

|  | **Date** | |  | |  |  |  |  |  |  |  |
| --- | --- | --- | --- | --- | --- | --- | --- | --- | --- | --- | --- |
|  | **Time** | |  | |  |  |  |  |  |  |  |
| **Criteria** | **Description** | **Score** | | | | | | | | | |
| **1. Alertness** | - Deeply asleep (eyes closed, no response to environmental stimuli) | **1** | |  |  |  |  |  |  |  |  |
|  | - Lightly asleep (eyes mostly closed, occasional responses) | **2** | |  |  |  |  |  |  |  |  |
|  | - Drowsy (eyes frequently closed, less responsive to the environment) | **3** | |  |  |  |  |  |  |  |  |
|  | - Awake and alert (child responsive to the environment) | **4** | |  |  |  |  |  |  |  |  |
|  | - Awake & hyper-alert (exaggerated responses to environmental stimuli) | **5** | |  |  |  |  |  |  |  |  |
| **2. Calmness/**  **Agitation** | - Calm (child appears serene and tranquil) | **1** | |  |  |  |  |  |  |  |  |
|  | - Slightly anxious (child shows slight anxiety) | **2** | |  |  |  |  |  |  |  |  |
|  | - Anxious (child appears agitated but remains in control) | **3** | |  |  |  |  |  |  |  |  |
|  | - Very anxious (child appears very agitated, just able to control) | **4** | |  |  |  |  |  |  |  |  |
|  | - Panicky (severe distress with loss of control) | **5** | |  |  |  |  |  |  |  |  |
| **3. Respiratory response**  (score only in mechanically  ventilated child) | - No spontaneous respiration | **1** | |  |  |  |  |  |  |  |  |
|  | - Spontaneous and ventilator respiration | **2** | |  |  |  |  |  |  |  |  |
|  | - Restlessness or resistance to ventilator | **3** | |  |  |  |  |  |  |  |  |
|  | - Actively breathes against ventilator or coughs regularly | **4** | |  |  |  |  |  |  |  |  |
|  | - Fights ventilator | **5** | |  |  |  |  |  |  |  |  |
| **Crying**  (score only in spontaneously  breathing child) | - Quiet breathing, no crying sounds | **1** | |  |  |  |  |  |  |  |  |
|  | - Occasional sobbing or moaning | **2** | |  |  |  |  |  |  |  |  |
|  | - Whining (monotonous sound) | **3** | |  |  |  |  |  |  |  |  |
|  | - Crying | **4** | |  |  |  |  |  |  |  |  |
|  | - Screaming or shrieking | **5** | |  |  |  |  |  |  |  |  |
| **4. Physical movement** | - No movement | **1** | |  |  |  |  |  |  |  |  |
|  | - Occasional, (three or fewer) slight movements | **2** | |  |  |  |  |  |  |  |  |
|  | - Frequent, (more than three) slight movements | **3** | |  |  |  |  |  |  |  |  |
|  | - Vigorous movements limited to extremities | **4** | |  |  |  |  |  |  |  |  |
|  | - Vigorous movements including torso and head | **5** | |  |  |  |  |  |  |  |  |
| **5. Muscle tone** | - Muscles totally relaxed; no muscle tone | **1** | |  |  |  |  |  |  |  |  |
|  | - Reduced muscle tone; less resistance than normal | **2** | |  |  |  |  |  |  |  |  |
|  | - Normal muscle tone | **3** | |  |  |  |  |  |  |  |  |
|  | - Increased muscle tone and flexion of fingers and toes | **4** | |  |  |  |  |  |  |  |  |
|  | - Extreme muscle rigidity and flexion of fingers and toes | **5** | |  |  |  |  |  |  |  |  |
| **6. Facial tension** | - Facial muscles totally relaxed | **1** | |  |  |  |  |  |  |  |  |
|  | - Normal facial tone | **2** | |  |  |  |  |  |  |  |  |
|  | - Tension evident in some facial muscles (not sustained) | **3** | |  |  |  |  |  |  |  |  |
|  | - Tension evident throughout facial muscles (sustained) | **4** | |  |  |  |  |  |  |  |  |
|  | - Facial muscles contorted and grimacing | **5** | |  |  |  |  |  |  |  |  |
|  | **Total Score** |  | |  |  |  |  |  |  |  |  |
|  | **Signature** |  | |  |  |  |  |  |  |  |  |

**Instructions for assessment by Comfort Behavioral Score (CBS):**

- Comfort B scoring (CBS) to be initiated for all patient
- Comfort B scoring (CBS) indication to be initiated by a physician order upon intubation, using NIV or for analgesia.
- The target score should be renewed/updated daily by a physician.
- Scoring should be done every 3 hours unless ordered otherwise by a physician.
- CBS indication to be modified by a physician order upon decision to wean sedation and/or analgesia.
- For patient on muscle relaxants, use CBS when the patient in drug- holiday period (give the patient a holiday from muscle relaxant. e.g hold the muscle relaxant every day at 8:00 a.m. for 30 minutes and during this time, do CBS).
- **CBS is not reliable alone and should be supplemented with subjective assessment in:** Patients with severe hypotonia or on muscle relaxants if drug holiday is contraindicated, brain death, patient weighing ≥50 kg.

**Patient’s criteria/Interpretation:**

**Is the patient intubated?** □ Yes □ No

**Goal of Assessment:**  □ Sedation (Intubated), refer to table (S3,1) & algorithm (A)

□ Sedation (NIV), refer to table (S3,1) & algorithm (A)

□ Analgesia refer to table (S3,2) & algorithm (B)

□ Routine screening of pain refer to (table S3,2)

|  | **Table S3,1: Sedation Cutoff Points** | | | |
| --- | --- | --- | --- | --- |
| **Comfort B**  **Score** | **6 – 10**  “Yellow area” | **11-15**  “Blue area” | **16- 22**  “Green area” | **23 – 30**  “Red area” |
| **Interpretation** | **Over sedation** | **Deep sedation** | **Moderate sedation** | **Under Sedation** |
| **Examples of Indications** | Burst suppression for refractory status epilepticus, critical airway such as fresh tracheostomy, or post TEF or airway repair ( 24-72 hrs) | Initiating NIV (initial 24 hrs), High ICP, high vent. settings, severe ARDS, critical pulm. HTN, High frequency ventilation | Maintaining NIV, low-moderate vent. settings | Pre-extubation |
| **Intervention**  ** If patient is receiving sedative, and is being monitored with CBS, then routine screening for pain is not applicable since sedatives and analgesia meds are overlapping in these patients (indicates no pain) | **If deep level of sedation is your target but score is sub-optimal**; 1- Give one IV bolus dose of sedative*, 2- Increase infusion rate by 20% (max. by 0.5 increment/ intervention), 3- Consider enteral chloral hydrate, 4- Reassess after 30-60 min  ***** If patient: **1-** is hemodynamically un-stable (significant hypotension or bradycardia), hold the infusion & ask the physician to assess. 2- had a previous side effect from the currently used sedatives, physician should assess before any intervention  **If lighter level of sedation is your target but score is sub-optimal**, 1- Decrease infusion rate by 20% (max. by 0.5 decrement/ intervention), after 30-60 min.2- Reassess after 30-60 min. | | | |
| **Sedatives Recommended Doses (Assuming normal Kidney and liver function)** | | | | |
| **Midazolam**** | **IV Bolus:** 0.05 mg/kg/dose (non-intubated) & 0.1 mg/kg/dose (intubated) (**max.** 2mg/dose)  **Continuous IV infusion:** 0.5-2 mcg/kg/min initially, titrate to desired effect. | | | |
| **Lorazepam**** | **IV /PO: :** 0.05 mg/kg/dose (non-intubated) and 0.1 mg/kg/dose (intubated) q4-8 hours (**max.** 2mg/dose) | | | |
| **Diazepam**** | **IV/PO: :** 0.05 mg/kg/dose (non-intubated) and 0.1 mg/kg/dose (intubated) q4-8 hours (**max.** 2mg/dose) | | | |
| **Precedex®** | **Continuous IV infusion:** 0.2-1 mcg/kg/hr | | | |
| **Ketamine** | **IV Bolus:** 0.5 mg/kg (non-intubated) & 1mg/kg (intubated) (**max.**2mg/kg) **Cont. IV infusion:** 5-20 mcg /kg/min | | | |

| **Table S3,2: Pain Cutoff Points** | | | |
| --- | --- | --- | --- |
| **Comfort B Score** | **6-17**  “Blue area” | **18-22**  “Green area” | **23 – 30**  “Red area” |
| **Interpretation** | **No pain** | **Mild- moderate pain** | **Severe pain** |
| **Intervention**  ** Reassess 30-60 min. after each intervention |  | - Consider reversible, non- pharmacological causes**^#^**.  - If score still in green area, inform PICU physician for full assessment.  - If pain is the most likely cause, consider the following sequentially:  **- Paracetamol**  **- Ibuprofen** if:  1-already not controlled by regular paracetamol  2-and/or high risk for pain such as post op or sickle cell crises.  **- Diclofenac suppositories**  OR  **- Paracetamol + Ibuprofen** | - Consider reversible, non- pharmacological causes**^#^**.  - If score still in red area, inform PICU physician for full assessment immediately.   - Schedule regular morphine boluses OR - Give one bolus dose of opioid (morphine or fentanyl) and start /increase infusion rate by 20% then reassess after 30-60 min - Consider PCA (patient Controlled Analgesia) - Consult pain management team. |
| **Analgesics Recommended Doses (Assuming normal Kidney and liver function)** | | | |
| **Paracetamol (Acetaminophen)** | **PO/PR:** 10-15 mg/kg dose q4-6h (**max.** 75mg/kg/day, or 3g/day)  **IV:** wt ≤10kg: 7.5 mg/kg q6h (**max.** 30 mg/kg/d). wt >10kg: 10- 15 mg/kg q6h (**max.** 60mg/kg/day, or 3g/day) | | |
| **Ibuprofen** | **PO:** 5-10 mg/kg dose q6-8h, **max.** 40mg/kg/day (Use is Not recommended if CrCl < 30 ml/min) | | |
| **Diclofenac** | **Rectal suppository:** 1 mg/kg/dose q 12 hr (**max.** 50mg/dose) (Use is Not recommended if CrCl < 30 ml/min) | | |
| **Fentanyl** | **IV Bolus:** 0.5 mcg/kg/dose (non-intubated) and 1 mcg/kg/dose (intubated) (**max.** 25mcg/dose)  **Continuous IV infusion**: 0.5-2 mcg/kg/hr initially, titrate to desired effect. | | |
| **Morphine** | **PO:** 0.1-0.5 mg/kg dose q4-6h/ **IV Bolus:** 0.05 mg/kg/dose (non-intubated) & 0.1 mg/kg/dose (intubated) q4-6 hrs  (**max.** 10 mg/dose) **Continuous IV infusion:** 10- 50 mcg/kg/hr titrate to desired effect. | | |

**^#^** **^Reversible, non- pharmacological causes can include:^** ^hunger, social (e.g. mother to be around), less handling, calm environment, noxious procedures, etc.^
