# Supplementary material for: Adapting Evidence‐Based Practice Guidelines for Sedation, Analgesia, Withdrawal, and Delirium Assessment and Management in Critically Ill Children
Source: Crit Care Res Pract. 2026 Jun 12;2026:7830579. doi: 10.1155/ccrp/7830579 (PMC13263535; doi:10.1155/ccrp/7830579)
Supplement: Supplementary file 1 — Supporting Information The Supporting Information provides the assessment tools, scoring systems, and implementation aids used in the adapted CPG. Supporting Tables S1–S9 include the PIPOH model guiding question formulation; AGREE II domain scores for the source guidelines; the Modified‐CBS; WAT‐1; risk categorization tables, dosing, and conversion thresholds for sedation and analgesia weaning; and the CAPD delirium assessment and management guide. These supporting files are intended to support the implementation of the adapted guideline in clinical practice. Supporting Table S1; Supporting Digital Content 1: Health/Clinical Questions (PIPOH Model) outlining the clinical questions that guided the adaptation process. Supporting Table S2; Supporting Digital Content 1: AGREE II standardized domain scores for sedation and analgesia for critically ill children in PICU; AGREE II standardized domain scores for each Source CPG included in the appraisal. Supporting Table S3: Modified‐CBS for pain and sedation assessment; used for assessing pain and sedation in critically ill children. Supporting Table S4: WAT‐1; used for monitoring opioid and benzodiazepine withdrawal symptoms. Supporting Table S5: Risk categories for withdrawal, including definitions and associated adverse outcomes. Supporting Table S6: (Weaning IV sedation/analgesia to conversion thresholds): Criteria for transitioning from IV sedation/analgesia to conversion thresholds during the weaning process. Supporting Table S7: Conversion of opioids and benzodiazepines from IV infusion to enteral; used to guide switching opioids and benzodiazepines from IV infusion to enteral formulations. Supporting Table S8: Lowest starting doses for PO agents after which frequency can be weaned: recommended lowest starting doses for oral agents to support safe and structured dose weaning. Supporting Table S9: Delirium assessment and management using CAPD score. Figure S1. Summary of the KSU‐modified ADAPTE process for CPG adaptat [file CCRP-2026-7830579-s001.zip › Table S4 WAT-1.docx]

**Table S4: WITHDRAWAL ASSESSMENT TOOL VERSION 1 (WAT – 1)**

© 2007 L.S. Franck and M.A.Q. Curley. All Rights reserved. Reproduced only by permission of Authors.

| **Patient identifier**  **Date:**  **Time:** | |  | | | | | | | | | | | | |
| --- | --- | --- | --- | --- | --- | --- | --- | --- | --- | --- | --- | --- | --- | --- |
|  |  |  |  |  |  |  |  |  |  |  |  |  |  |  |
|  |  |  |  |  |  |  |  |  |  |  |  |  |  |  |
| ***Information from patient record, previous 12 hours*** | | | | | | | | | | | | | | |
| **Any loose /watery stools** No = 0  Yes = 1 | |  |  |  |  |  |  |  |  |  |  |  |  |  |
| **Any vomiting/wretching/gagging** No = 0  Yes = 1 | |  |  |  |  |  |  |  |  |  |  |  |  |  |
| **Temperature > 37.8^o^ C** No = 0  Yes = 1 | |  |  |  |  |  |  |  |  |  |  |  |  |  |
| ***2 minute pre-stimulus observation*** | | | | | | | | | | | | | | |
| **State** CBS^1^ ≤22 or asleep/awake/calm = 0  CBS^1^ ≥23 or awake/distressed = 1 | |  |  |  |  |  |  |  |  |  |  |  |  |  |
| **Tremor** None/mild = 0  Moderate/severe = 1 | |  |  |  |  |  |  |  |  |  |  |  |  |  |
| **Any sweating** No = 0  Yes = 1 | |  |  |  |  |  |  |  |  |  |  |  |  |  |
| **Uncoordinated/repetitive movement**  None/mild = 0  Moderate/severe = 1 | |  |  |  |  |  |  |  |  |  |  |  |  |  |
| **Yawning or sneezing**  None or 1 = 0  >2 = 1 | |  |  |  |  |  |  |  |  |  |  |  |  |  |
| ***1 minute stimulus observation*** | | | | | | | | | | | | | | |
| **Startle to touch** None/mild = 0  Moderate/severe = 1 | |  |  |  |  |  |  |  |  |  |  |  |  |  |
| **Muscle tone** Normal = 0  Increased = 1 | |  |  |  |  |  |  |  |  |  |  |  |  |  |
| ***Post-stimulus recovery*** | | | | | | | | | | | | | | |
| < 2min = 0  **Time to gain calm state (CBS^1^ ≤22)** 2 - 5min = 1  > 5 min = 2 | |  |  |  |  |  |  |  |  |  |  |  |  |  |
| **Total Score (0-12)** | |  |  |  |  |  |  |  |  |  |  |  |  |  |
| **Mean Score** |  | **Worst Score** | | | | | | |  | | | | | |

**WITHDRAWAL ASSESSMENT TOOL (WAT – 1) INSTRUCTIONS**

- Start WAT-1 scoring from the first day of weaning in patients who have received opioids +/or benzodiazepines by infusion or regular dosing for prolonged periods (e.g., > 5 days). Continue twice daily scoring until 72 hours after the last dose.
- The Withdrawal Assessment Tool (WAT-1) should be completed along with the CBS^1^ at least once per 12 hour shift (e.g., at 08:00 and 20:00 ± 2 hours). ****The progressive stimulus:** use a calm voice, call the patient’s name **→**Call the patient’s name and gently touch the patient’s body →Asses the patient’s response to a planned noxious procedure, e.g., ET suctioning → If a noxious procedure is not planned then, use a pencil/pen, provide < 5 seconds of direct pressure to the patient’s nail bed.
- **Obtain information from patient record (this can be done before or after the progressive stimulus^**^):**
- **Loose/watery stools:** Score 1 if any loose or watery stools were documented in the past 12 hours; score 0 if none were noted.
- **Vomiting/wretching/gagging:** Score 1 if any vomiting or spontaneous wretching or gagging were documented in the past 12 hours; score 0 if none were noted
- **Temp. > 37.8^o^ C:** Score 1 if the most frequently occurring temp. Documented was >37.8^o^C in the past 12 hours; score 0 if this was not the case.

**2 minute pre-stimulus observation:**

- **State:** Score 1 if awake and distress (CBS^1^: ≥ 23) observed during the 2 minutes prior to the stimulus; score 0 if asleep or awake and calm/cooperative (CBS^1^≤ 22).
- **Tremor:** Score 1 if moderate to severe tremor observed during the 2 minutes prior to the stimulus; score 0 if no tremor (or only minor, intermittent tremor).
- **Sweating:** Score 1 if any sweating during the 2 minutes prior to the stimulus; score 0 if no sweating noted.
- **Uncoordinated/repetitive movements:** Score 1 if moderate to severe uncoordinated or repetitive movements such as head turning, leg or arm flailing or torso arching observed during the 2 minutes prior to the stimulus; score 0 if no (or only mild) uncoordinated or repetitive movements.
- **Yawning or sneezing** **> 1:** Score 1 if more than 1 yawn or sneeze observed during the 2 min. prior to the stimulus; score 0 if 0 to 1 yawn or sneeze.

**1 minute stimulus observation:**

- **Startle to touch:** Score 1 if moderate to severe startle occurs when touched during the stimulus; score 0 if none (or mild).
- **Muscle tone:** Score 1 if tone increased during the stimulus; score 0 if normal.

**Post-stimulus recovery:**

- **Time to gain calm state** (CBS^1^≤ 22): Score 2 if it takes greater than 5 minutes following stimulus; score 1 if achieved within 2 to 5 minutes; score 0 if achieved in less than 2 minutes.
- **Sum the 11 numbers in the column for the total WAT-1 score (0-12).**

1: CBS (modified Comfort Behavioral Scale for pain and sedation assessment, KSUMC, PICU

- **(WAT – 1) Score Interpretation:**
- A higher WAT-1 score indicates more withdrawal symptoms while a lower score indicates fewer.
- Available evidence identifies iatrogenic withdrawal as a WAT-1 Score of ≥ 3.
- **Management according to score:**

**# Long acting drugs:** Morphine or Methadone if opioids withdrawal. Lorazepam or Diazepam if benzodiazepines withdrawal.

| **Withdrawal Management Drugs and Recommended Doses** | |
| --- | --- |
| **Lorazepam** | **IV Bolus:** 0.05- 0.1 mg/kg/dose (Slow IV push over 2-5 min) q4-8 hours (**max.** 2mg)  **PO:** 0.05- 0.1 mg/kg/dose q4-8 hours (**max.** 2mg) |
| **Diazepam** | **IV Bolus:** 0.05- 0.1 mg/kg/dose (Slow IV push over 2-5 min) q4-8 hours (**max.** 2mg)  **PO:** 0.05- 0.1 mg/kg/dose q4-8 hours (**max.** 2mg) |
| **Morphine** | **IV Bolus:** 0.05- 0.1 mg/kg/dose (Slow IV push over 5 min) q4-6h (**max.** 10mg)  **PO:** 0.1-0.5 mg/kg dose q4-6h (**max.** 10mg) |
| **Methadone** | **IV Bolus:** 0.05- 0.1 mg/kg/dose (Slow IV push over 5 min) q4-6h (**max.** 10mg)  **PO:** 0.1- 0.2 mg/kg dose q4-6h (**max.** 10mg) |
| **Clonidine** | **PO:** 2-5 mcg/kg/dose q6-8h (**max.** 25mcg/kg/day or 0.9 mg/day)  Clonidine should not be weaned until opioids and benzodiazepines are stopped. When weaning Clonidine, reduce the dose by 10% - 20% every 24 hrs. If a patient has been receiving clonidine for more than 1 week, weaning should take place over at least 5 days.  Once the patient has reached a dose of 0.5 mcg/kg reduce the frequency to 8 hourly for 24 hours then 12 hourly for 24 hours, then once daily for 24 hours and then stop. |
