# Supplementary material for: Adapting Evidence‐Based Practice Guidelines for Sedation, Analgesia, Withdrawal, and Delirium Assessment and Management in Critically Ill Children
Source: Crit Care Res Pract. 2026 Jun 12;2026:7830579. doi: 10.1155/ccrp/7830579 (PMC13263535; doi:10.1155/ccrp/7830579)
Supplement: Supplementary file 1 — Supporting Information The Supporting Information provides the assessment tools, scoring systems, and implementation aids used in the adapted CPG. Supporting Tables S1–S9 include the PIPOH model guiding question formulation; AGREE II domain scores for the source guidelines; the Modified‐CBS; WAT‐1; risk categorization tables, dosing, and conversion thresholds for sedation and analgesia weaning; and the CAPD delirium assessment and management guide. These supporting files are intended to support the implementation of the adapted guideline in clinical practice. Supporting Table S1; Supporting Digital Content 1: Health/Clinical Questions (PIPOH Model) outlining the clinical questions that guided the adaptation process. Supporting Table S2; Supporting Digital Content 1: AGREE II standardized domain scores for sedation and analgesia for critically ill children in PICU; AGREE II standardized domain scores for each Source CPG included in the appraisal. Supporting Table S3: Modified‐CBS for pain and sedation assessment; used for assessing pain and sedation in critically ill children. Supporting Table S4: WAT‐1; used for monitoring opioid and benzodiazepine withdrawal symptoms. Supporting Table S5: Risk categories for withdrawal, including definitions and associated adverse outcomes. Supporting Table S6: (Weaning IV sedation/analgesia to conversion thresholds): Criteria for transitioning from IV sedation/analgesia to conversion thresholds during the weaning process. Supporting Table S7: Conversion of opioids and benzodiazepines from IV infusion to enteral; used to guide switching opioids and benzodiazepines from IV infusion to enteral formulations. Supporting Table S8: Lowest starting doses for PO agents after which frequency can be weaned: recommended lowest starting doses for oral agents to support safe and structured dose weaning. Supporting Table S9: Delirium assessment and management using CAPD score. Figure S1. Summary of the KSU‐modified ADAPTE process for CPG adaptat [file CCRP-2026-7830579-s001.zip › Table S5,S6,S7,S81.docx]

**Table S5: Risk Categories for withdrawal and withdrawal associated adverse outcomes**

| **Low risk** | - Total duration of sedation and analgesia is less than 5 days and within standard drug dose ranges.  - Little to no risk of adverse events if withdrawal symptoms occur (no high risk criteria present). |
| --- | --- |
| **Moderate risk** | - Total duration of sedation and analgesia is more than 5 days but less than 9 days OR  - Less than 5 days and above standard drug dose ranges. |
| **High risk** | - Total duration of sedation and analgesia >9 days (regardless of the dose)  - Cumulative dose for fentanyl >2.5mg/kg and Midazolam >60mg/kg.  - Increased risk of adverse events if withdrawal symptoms occur (if have those criteria);   - Seizure disorders - Hemodynamically significant congenital heart disease - Pulmonary hypertension - Prior history of weaning difficulty or complications from withdrawal |

**Table S6: Weaning IV Sedation/analgesia to Conversion Thresholds**

- Titrate opioids and BDz drips down to target doses before starting wean plans (IV or Enteral).
- These doses can safely be converted to enteral agents based on available medication strengths, formulations.

| **Drug** | **Goal Threshold of IV Continuous Infusions to convert to Enteral** |
| --- | --- |
| Fentanyl | ≤ 2 mcg/kg/hr |
| Midazolam | ≤ 1.5 mcg/kg/min |
| Morphine | ≤ 0.04 mg/kg/hr (≤40 mcg/kg/hr) |

**Table S7: Conversion of opioids and BDZs from IV infusion to Enteral:**

| **Continuous Medication Infusion** | **Enteral Agent (PO/GT/JT/NG/NJ/ND)** |
| --- | --- |
| Fentanyl….mcg/kg/hr | **Calculate enteral morphine equivalent**  Fentanyl ___ mcg/kg/hr x___ kg x 0.05 = ___mg enteral **morphine**/dose  Calculated enteral **morphine** dose should be given q6h  **Convert to desired enteral agent if other than enteral morphine**  Enteral morphine to Enteral methadone  ___ mg enteral morphine/dose x 0.25 = ___ mg enteral **methadone**/dose  Calculated enteral **methadone** dose should be given q 6h |
| Midazolam….mcg/kg/min | Midazolam___ mcg/kg/min x ___kg x 0.05 = ___mg enteral **diazepam**/dose  Calculated enteral **diazepam** dose should be given q6h  Midazolam ___ mcg/kg/min x ___kg x 0.025 =___ mg enteral **lorazepam**/dose  Calculated enteral **lorazepam** dose should be given q6h |

**Table S8: Lowest Starting Doses for PO Agents after which Frequency can be weaned:**

| **Drug** | **Lowest starting dose (<50kg) mg/kg PO** | **Lowest starting dose (≥50kg) mg PO** |
| --- | --- | --- |
| **Opioids** | | |
| Morphine | 0.15 mg/kg/dose PO | 7.5mg PO |
| Methadone | 0.05 mg/kg/dose PO | 2.5mg PO |
| **Benzodiazepines** | | |
| Diazepam | 0. 05mg/kg/dose PO | 2.5mg PO |
| Lorazepam | 0. 05mg/kg/dose PO | 2.5mg PO |
