# Supplementary material for: Adapting Evidence‐Based Practice Guidelines for Sedation, Analgesia, Withdrawal, and Delirium Assessment and Management in Critically Ill Children
Source: Crit Care Res Pract. 2026 Jun 12;2026:7830579. doi: 10.1155/ccrp/7830579 (PMC13263535; doi:10.1155/ccrp/7830579)
Supplement: Supplementary file 1 — Supporting Information The Supporting Information provides the assessment tools, scoring systems, and implementation aids used in the adapted CPG. Supporting Tables S1–S9 include the PIPOH model guiding question formulation; AGREE II domain scores for the source guidelines; the Modified‐CBS; WAT‐1; risk categorization tables, dosing, and conversion thresholds for sedation and analgesia weaning; and the CAPD delirium assessment and management guide. These supporting files are intended to support the implementation of the adapted guideline in clinical practice. Supporting Table S1; Supporting Digital Content 1: Health/Clinical Questions (PIPOH Model) outlining the clinical questions that guided the adaptation process. Supporting Table S2; Supporting Digital Content 1: AGREE II standardized domain scores for sedation and analgesia for critically ill children in PICU; AGREE II standardized domain scores for each Source CPG included in the appraisal. Supporting Table S3: Modified‐CBS for pain and sedation assessment; used for assessing pain and sedation in critically ill children. Supporting Table S4: WAT‐1; used for monitoring opioid and benzodiazepine withdrawal symptoms. Supporting Table S5: Risk categories for withdrawal, including definitions and associated adverse outcomes. Supporting Table S6: (Weaning IV sedation/analgesia to conversion thresholds): Criteria for transitioning from IV sedation/analgesia to conversion thresholds during the weaning process. Supporting Table S7: Conversion of opioids and benzodiazepines from IV infusion to enteral; used to guide switching opioids and benzodiazepines from IV infusion to enteral formulations. Supporting Table S8: Lowest starting doses for PO agents after which frequency can be weaned: recommended lowest starting doses for oral agents to support safe and structured dose weaning. Supporting Table S9: Delirium assessment and management using CAPD score. Figure S1. Summary of the KSU‐modified ADAPTE process for CPG adaptat [file CCRP-2026-7830579-s001.zip › Table S9 Delirium Assessment and management (1).docx]

**Table S9: Delirium Assessment and Management using Cornell Assessment of Pediatric Delirium (CAPD) Score**

**Instructions:**

- Always apply preventive measures.
- Screen for Delirium Twice Daily with CAPD Scale.

**Preventive measures:**

- Establish daily routines and schedules (cluster care at night, sleep hygiene, doors closed with lights, TV and others should be off while asleep, control light and noise in the patient room).
- Re-orient patient to time and place.
- Promote a familiar environment (toys, identify consistent caregivers and promote parental involvement, use needed adaptive equipment and/or communication aids (e.g. glasses/hearing aids))
- Minimize/avoid use of restraints.
- Daily review of need for tubes/lines.
- Encourage early mobilization as appropriate.

**Types of Delirium:**

| **Type** | **Clinical Manifestations** | **Risk for Self-Harm** | **Immediate Recommendations** |
| --- | --- | --- | --- |
| **Hypoactive** | Non-interactive, sleepy, comatose | - Low | - Identify cause and treat. - Continue prevention measures. |
| **Hyperactive** | Agitated, excitable | - May be a risk to self | - Identify cause and treat. - Continue prevention measures. - Consider trial of pharmacological Therapy |
| **Mixed** | A state in which the patient alternates between hypo- and hyperactive delirium. |  |  |

**Screening:**

**Step 1: Arousal Assessment using** **Comfort B scoring (CBS)**

- Do CBS to assess level of arousal.
- If the score lies in the yellow or blue area, stop and reassess the patient later.
- If the score lies in green or red area, proceed to **step 2**.
- For children who are not receiving sedatives, CBS score in blue area is acceptable to proceed to **step 2**.

|  | **Table 1,1: Sedation Cutoff Points** | | | |
| --- | --- | --- | --- | --- |
| **Comfort B**  **Score** | **6 – 10**  “Yellow area” | **11-15**  “Blue area” | **16- 22**  “Green area” | **23 – 30**  “Red area” |
| **Interpretation** | **Over sedation** | **Deep sedation** | **Moderate sedation** | **Under Sedation** |
| **Examples of Indications** | Burst suppression for refractory status epilepticus, critical airway such as fresh tracheostomy, or post TEF or airway repair ( 24-72 hrs) | Initiating NIV (initial 24 hrs), High ICP, high vent. settings, severe ARDS, critical pulm. HTN, High frequency ventilation | Maintaining NIV, low-moderate vent. settings | Pre-extubation |

**Step 2: Content Assessment using** **CAPD**

| **Please answer the following questions based on your interactions with the patient over the course of your shift:** | **Never 4** | **Rarely 3** | **Sometimes 2** | **Often 1** | **Always 0** | **Score** |
| --- | --- | --- | --- | --- | --- | --- |
| 1. Does the child make eye contact with the caregiver? |  |  |  |  |  |  |
| 2. Are the child's actions purposeful? |  |  |  |  |  |  |
| 3. Is the child aware of his/her surroundings? |  |  |  |  |  |  |
| 4. Does the child communicate needs and wants? |  |  |  |  |  |  |
|  | **Never 0** | **Rarely 1** | **Sometimes 2** | **Often 3** | **Always 4** | **Score** |
| 5. Is the child restless? |  |  |  |  |  |  |
| 6. Is the child inconsolable? |  |  |  |  |  |  |
| 7. Is the child underactive — very little movement while awake? |  |  |  |  |  |  |
| 8. Does it take the child a long time to respond to interactions? |  |  |  |  |  |  |
| **TOTAL** |  |  |  |  |  |  |


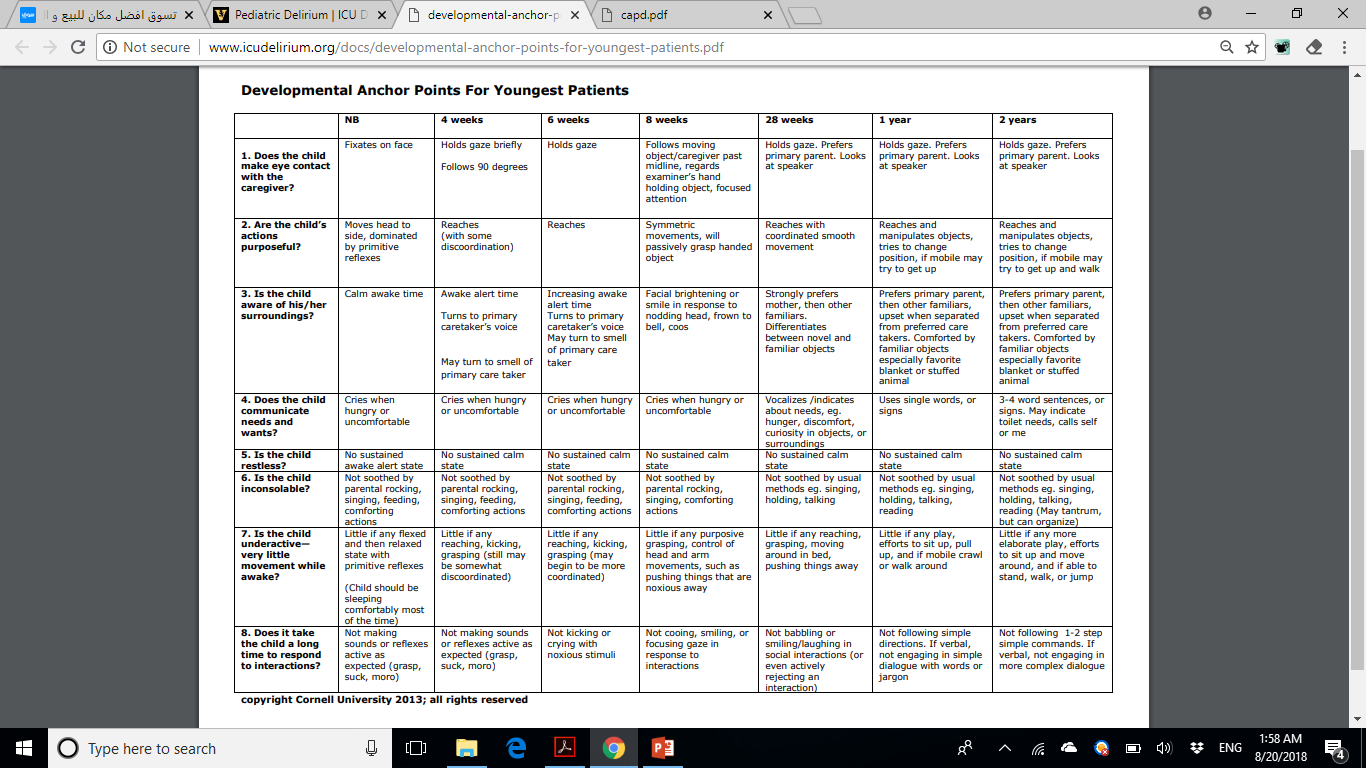
**Developmental Anchors to Guide Use of CAPD Tool for Patients < 2 Years:**

**Step 3: CAPD Interpretation:**

| **CAPD Screen** | **Value** | **Comments** |
| --- | --- | --- |
| **Positive** | **≥ 9 in developmentally appropriate patient** | - Evaluate patient for delirium and other causes of acute agitation or hypo activity using **BRAIN MAPS**. - Modify environment, evaluate and consider discontinuing medications that contribute to delirium. - Treat underlying illness. - If delirium persists or patient is a risk of self-harm, consider a trial of pharmacological therapy ([Dexmedetomidine](http://www.crlonline.com/lco/action/doc/retrieve/docid/chiphi_f/788050), typical or atypical antipsychotics). - Assess for Resolution and continue preventative measures - Continue CAPD screening twice Daily while in PICU. - If pharmacologic therapy started, evaluate ability to discontinue no later than 5-7 days post-initiation. - Patients who are not at risk to themselves but are on pharmacologic therapy can be managed outside of the ICU in consultation with psychiatry without the need to continue twice daily CAPD screening. |
|  | **≥ 9 in developmentally delayed patient** | - A trended score of > 9 with a parental assessment that the patient’s activity is different than normal should raise concern for delirium. - Treat underlying illness. - A trended score > 9 but with normal activity per parent report should not be considered delirious. - Consider consult to Psychiatry with/out Behavioral Pediatrics for evaluation of this subpopulation. |
| **Negative** | **< 9** | Continue to screen twice daily and apply preventive measures. |


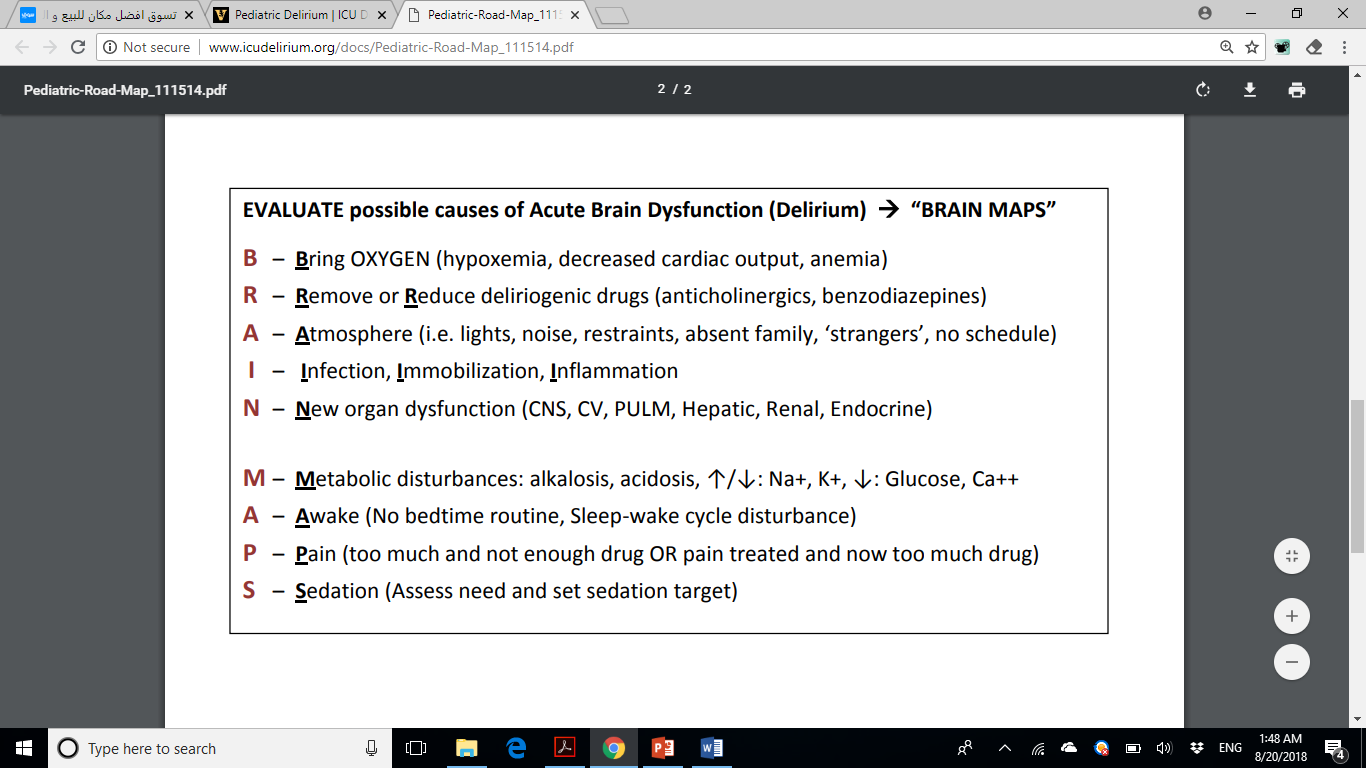


**Management of Delirium:**

| **Pharmacological interventions (** **Consult pediatric psychiatry before starting any therapy)** | | | |
| --- | --- | --- | --- |
| **Type** | **Drug** | **Dose** | **Monitoring and side effects** |
| **Hypoactive or mixed** | **Risperidone**  **(2^nd^ generation)** | **Children <5 years: Oral:** Initial: 0.1 to 0.2 mg once daily at bedtime.  **Children ≥5 years and Adolescents:** Oral: Initial: 0.2 to 0.5 mg once daily at bedtime; may titrate to lowest effective dose every 1 to 2 days; usual range: 0.2 to 2.5 mg/day in divided doses 2 to 4 times daily; maximum daily dose dependent upon patient weight: <20 kg: 1 mg/day; 20 to 45 kg: 2.5 mg/day, >45 kg: 3 mg/day | EPS, ECG, anticholinergic effects, CBC, baseline lipid panel, weight, glucose, NMS |
|  | **Quetiapine**  **(1^st^  generation)** | **Oral:** 1.3- 1.7 mg/kg/day  As-needed doses of 0.5 mg/kg/dose were given for breakthrough delirium (no more than Q6hr) | BBW (suicidal thoughts and behavior), anticholinergic effects, baseline lipid panel, weight, EPS, glucose, NMS |
| **Hyperactive** | **Haloperidol**  **(2^nd^ generation)** | **Infants ≥3 months, Children, and Adolescents: IV (lactate, immediate release):** Loading dose: 0.15 to 0.25 mg/dose infused slowly over 30 to 45 minutes; maintenance dose: 0.05 to 0.5 mg/kg/day in divided doses (every 6-12hr) | EPS, ECG, anticholinergic effects, CBC, NMS |
|  | **Olanzapine**  **(2^nd^ generation)** | **Children <4 years:** 0.625 mg once to twice daily  **Children 4- <6 years:** 1.25 mg once to twice daily  **Children ≥6 years:** 2.5–5 mg once to twice daily | BBW (post injection delirium/sedation syndrome), ECG, CBC, anticholinergic effects, baseline lipid panel, weight, EPS, glucose, NMS |

**Abbreviations: BBW:** Black box warning, **ECG:** Electrocardiogram, **EPS:** Extrapyramidal side effects, **NMS:** Neuroleptic malignant syndrome.

| **Non pharmacological interventions** | |
| --- | --- |
| **Optimize patient’s environment** | - Reduce noise, avoid physical restraints, and minimize staff changes - Increase light during the day and dim the lights at night - Use pictures of family, familiar toys - Avoid triggers - Keep a constant schedule |
| **Daily reorientation** | - Orient the patient to the day, time, place and situation - May use clocks and calendars in patient’s room - Sedation holidays |
| **Reduce utilization of** **deliriogenic medications (e.g. benzodiazepines, ketamine)** | Regular pain and sedation score assessment using CBS to eliminate unnecessary use.  Consider using dexmedetomidine as sedative/analgesic. |
