# Supplementary material for: Adapting Evidence‐Based Practice Guidelines for Sedation, Analgesia, Withdrawal, and Delirium Assessment and Management in Critically Ill Children
Source: Crit Care Res Pract. 2026 Jun 12;2026:7830579. doi: 10.1155/ccrp/7830579 (PMC13263535; doi:10.1155/ccrp/7830579)
Supplement: Supplementary file 1 — Supporting Information The Supporting Information provides the assessment tools, scoring systems, and implementation aids used in the adapted CPG. Supporting Tables S1–S9 include the PIPOH model guiding question formulation; AGREE II domain scores for the source guidelines; the Modified‐CBS; WAT‐1; risk categorization tables, dosing, and conversion thresholds for sedation and analgesia weaning; and the CAPD delirium assessment and management guide. These supporting files are intended to support the implementation of the adapted guideline in clinical practice. Supporting Table S1; Supporting Digital Content 1: Health/Clinical Questions (PIPOH Model) outlining the clinical questions that guided the adaptation process. Supporting Table S2; Supporting Digital Content 1: AGREE II standardized domain scores for sedation and analgesia for critically ill children in PICU; AGREE II standardized domain scores for each Source CPG included in the appraisal. Supporting Table S3: Modified‐CBS for pain and sedation assessment; used for assessing pain and sedation in critically ill children. Supporting Table S4: WAT‐1; used for monitoring opioid and benzodiazepine withdrawal symptoms. Supporting Table S5: Risk categories for withdrawal, including definitions and associated adverse outcomes. Supporting Table S6: (Weaning IV sedation/analgesia to conversion thresholds): Criteria for transitioning from IV sedation/analgesia to conversion thresholds during the weaning process. Supporting Table S7: Conversion of opioids and benzodiazepines from IV infusion to enteral; used to guide switching opioids and benzodiazepines from IV infusion to enteral formulations. Supporting Table S8: Lowest starting doses for PO agents after which frequency can be weaned: recommended lowest starting doses for oral agents to support safe and structured dose weaning. Supporting Table S9: Delirium assessment and management using CAPD score. Figure S1. Summary of the KSU‐modified ADAPTE process for CPG adaptat [file CCRP-2026-7830579-s001.zip › Table S1 (PIPOH Model).doc]

# Table S1: Health/ Clinical Questions (PIPOH Model)

| **P: Patient (Target Population):** | All children aged 1 month to 14 years admitted to the PICU and required non-invasive ventilation, invasive mechanical ventilation, or post-operative analgesia, specific procedures and started on intermittent or continuous infusions of sedatives and/ or analgesics. |
| --- | --- |
| **I: Interventions and Practices Considered/ CPG Category:** | Sedation and analgesia initiation, adjustment, titration, weaning, and assessment. |
| **P: Professionals (Intended / Target Users or Stakeholders)/ Clinical specialties**: | All physicians, nurses, pharmacists working in the PICU |
| **O: Major Outcomes Considered:** | - PICU patient population exposure to opioids and benzodiazepines (cumulative doses, total duration). - Percentage of achieved targeted CBS and percentage of patients with WAT-1 score <3. - Mechanical ventilation free days in the PICU. - PICU length of stay. - Compliance of PICU Medical Team in implementing the current the CPG |
| **H: Healthcare Settings:** | Pediatric Intensive Care Units (PICUs) |
